# Bis(μ-3,5-dinitro-2-oxidobenzoato)-κ^3^ *O* ^1^,*O* ^2^:*O* ^1^;κ^3^ *O* ^1^:*O* ^1^,*O* ^2^-bis[aqua­(2-phenyl-1,3,7,8-tetra­azacyclo­penta­[*l*]phenanthrene-κ^2^ *N* ^7^,*N* ^8^)cobalt(II)]

**DOI:** 10.1107/S1600536810017629

**Published:** 2010-06-05

**Authors:** Xiang-Cheng Wang, Jing Chen, Chun-Jie Wang, Chun-Xiang Li

**Affiliations:** a School of Chemistry and Chemical Engineering, Jiangsu University, Zhenjiang 212013, People’s Republic of China

## Abstract

In the title compound, [Co_2_(C_7_H_2_N_2_O_7_)_2_(C_19_H_12_N_4_)_2_(H_2_O)_2_], the Co^II^ atom is six-coordinated by two N atoms from a 2-phenyl-1*H*-1,3,7,8,-tetraaza­cyclo­penta­[*l*]phenanthrene (*L*) ligand, three O atoms from two 3,5-dinitro-2-oxidobenzoate (3,5-dinitro­salicylate or DNSA) ligands and one O atom from a water mol­ecule in a distorted octa­hedral geometry. The Co^II^ atoms are bridged by two carboxyl­ate O atoms from two DNSA ligands, forming a centrosymmetric dinuclear structure. Neighbouring dinuclear units inter­act with each other through two types of π–π inter­actions between the *L* ligands [shortest centroid–centroid distance = 3.646 (3) Å] and between the *L* and DNSA ligands [shortest atom-to-centroid distance = 3.794 (3) Å]. N—H⋯O, O—H⋯N and O—H⋯O hydrogen bonds are observed, which lead to a three-dimensional structure.

## Related literature

For general background to metal–organic coordination polymers, see: Che *et al.* (2008 ▶). For a related structure, see: Liu *et al.* (2009 ▶). For the ligand synthesis, see: Steck & Day (1943 ▶).
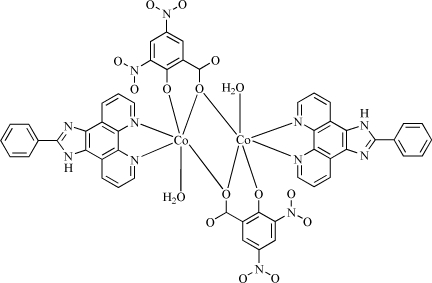


## Experimental

### 

#### Crystal data

[Co_2_(C_7_H_2_N_2_O_7_)_2_(C_19_H_12_N_4_)_2_(H_2_O)_2_]
*M* *_r_* = 1198.76
Triclinic, 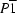

*a* = 8.2943 (4) Å
*b* = 11.0232 (5) Å
*c* = 13.6139 (7) Å
α = 102.690 (4)°
β = 107.282 (4)°
γ = 90.459 (4)°
*V* = 1155.9 (1) Å^3^
*Z* = 1
Mo *K*α radiation
μ = 0.81 mm^−1^
*T* = 292 K
0.32 × 0.27 × 0.23 mm

#### Data collection

Oxford Diffraction Gemini R Ultra CCD diffractometer
Absorption correction: multi-scan (*CrysAlis RED*; Oxford Diffraction, 2006 ▶) *T*_min_ = 0.771, *T*_max_ = 0.829
7153 measured reflections
4041 independent reflections
2933 reflections with *I* > 2σ(*I*)
*R*_int_ = 0.028

#### Refinement

*R*[*F* ^2^ > 2σ(*F* ^2^)] = 0.044
*wR*(*F* ^2^) = 0.111
*S* = 1.00
4041 reflections
378 parameters
168 restraints
H atoms treated by a mixture of independent and constrained refinement
Δρ_max_ = 0.97 e Å^−3^
Δρ_min_ = −1.00 e Å^−3^

### 

Data collection: *CrysAlis CCD* (Oxford Diffraction, 2006 ▶); cell refinement: *CrysAlis RED* (Oxford Diffraction, 2006 ▶); data reduction: *CrysAlis RED*; program(s) used to solve structure: *SHELXTL* (Sheldrick, 2008 ▶); program(s) used to refine structure: *SHELXTL*; molecular graphics: *SHELXTL* and *DIAMOND* (Brandenburg, 1999 ▶); software used to prepare material for publication: *SHELXTL*.

## Supplementary Material

Crystal structure: contains datablocks global, I. DOI: 10.1107/S1600536810017629/hy2303sup1.cif

Structure factors: contains datablocks I. DOI: 10.1107/S1600536810017629/hy2303Isup2.hkl

Additional supplementary materials:  crystallographic information; 3D view; checkCIF report

## Figures and Tables

**Table 1:** Selected bond lengths (Å)

Co—N1	2.102 (3)
Co—N2	2.095 (3)
Co—O1	2.050 (2)
Co—O1^i^	2.216 (3)
Co—O3	1.991 (3)
Co—O*W*1	2.139 (3)

Symmetry code: (i) 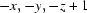.

**Table 2:** Hydrogen-bond geometry (Å, °)

*D*—H⋯*A*	*D*—H	H⋯*A*	*D*⋯*A*	*D*—H⋯*A*
N4—H4⋯O6^ii^	0.86	2.17	2.948 (5)	150
O*W*1—H1*WA*⋯N3^iii^	0.81 (6)	2.03 (6)	2.831 (5)	172 (5)
O*W*1—H1*WB*⋯O2^iv^	0.80 (5)	1.88 (5)	2.662 (4)	165 (5)

Symmetry codes: (ii) 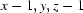; (iii) 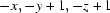; (iv) 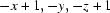.

**Fig. 1.**
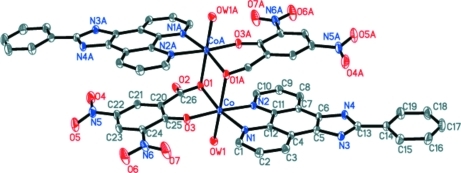
Molecular structure of the title compound. Displacement ellipsoids are drawn at the 30% probability level. H atoms have been omitted for clarity. [Symmetry code: (A) -x, -y, -z+1.]

**Fig. 2.**
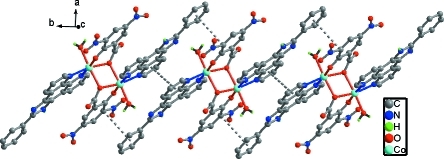
View of the one-dimensional supramolecular chain generated by π–π interactions (dashed lines). H atoms except those of water molecules have been omitted for clarity.

**Fig. 3.**
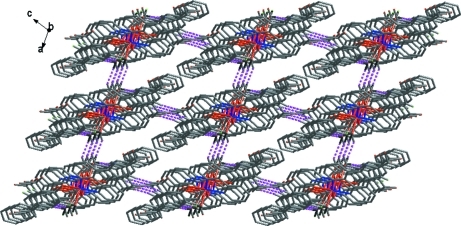
View of a three-dimensional supramolecular structure with hydrogen bonds indicated by dotted lines. Most H atoms have been omitted.

## supplementary crystallographic information

### Comment

Metal-organic coordination polymers have attracted increasing interest over the past decade because of their intriguing structures and tremendous potential applications in catalysis, molecular adsorption, nonlinear optics, magnetism, and so on (Che *et al.*, 2008). 1,10-Phenanthroline (phen) has been widely used to build supramolecular architectures owing to its excellent coordinating ability and large conjugated system. However, building blocks derived from the appropriate modification of phen, such as 2-phenyl-1*H*-1,3,7,8-tetra-azacyclopenta[l]phenanthrene (*L*) have received considerably less attention (Liu *et al.*, 2009). Hereby, we have prepared the title compound based on *L* and 3,5-dinitrosalicylic acid (H_2_DNSA) ligands.

In the title compound, the Co^II^ atom is six-coordinated by two N atoms from one *L* ligand, four O atoms from two DNSA ligands and one water molecule (Fig. 1). The Co—O distances range from 1.991 (3) to 2.216 (3) Å and the Co—N lengths are 2.095 (3) and 2.102 (3) Å (Table 1). The N1, N2, O1, O3 atoms comprise the equatorial plane, while the O1^i^ and OW1 atoms occupy the axial position [symmetry code: (i) -x, -y, 1-z]. A carboxylate O atom and the hydroxy O atom of the DNSA ligand coordinate to one Co atom, and this carboxylate O atom bridges the other Co atom, forming a dinuclear structure. The two nitro groups are uncoordinated.

It is noteworthy that various hydrogen bonds interactions are observed in the title compound. (a) An N—H···O hydrogen bond between the imidazole ring donor and the nitro group of the DNSA ligand (Table 2). (b) O—H···N or O—H···O hydrogen bonds involving the coordinated water molecule OW1 and the imidazole N3 and carboxylate O2 atoms (Table 2). In addition, two types of π–π stacking interactions further intensify the architectures. One is the offset face-to-face π–π interactions between the *L* ligands with the shortest centroid–centroid distance of 3.646 (3) Å, while the other exists between the *L* and DNSA ligands [shortest atom-to-centroid distance = 3.794 (3) Å] (Fig. 2), which lead to a three-dimensional supramolecular structure (Fig. 3).

### Experimental

The *L* ligand was synthesized according to the literature method (Steck & Day, 1943). The title compound was synthesized under hydrothermal conditions. A mixture of *L* (0.06 g, 0.2 mmol), H_2_DNSA (0.048 g, 0.2 mmol), Co(NO_3_)_2_ (0.036 g, 0.2 mmol) and water (10 ml) in a mole ratio 1:1:1:5000 was placed in a 25 ml Teflon-lined autoclave and heated for 3 d at 433 K under autogenous pressure. Upon cooling and opening the bomb, yellow block-shaped crystals were obtained, washed with H_2_O and dried in air (yield 45% based on Co).

### Refinement

H atoms on C and N atoms were positioned geometrically and refined as riding atoms, with C—H = 0.93 and N—H = 0.86 Å and with *U*_iso_(H) = 1.2*U*_eq_(C,N). H atoms of water molecule were located from a difference Fourier map and their positions and *U*_iso_ values were refined freely.

### Crystal data

**Table:**

[Co_2_(C_7_H_2_N_2_O_7_)_2_(C_19_H_12_N_4_)_2_(H_2_O)_2_]	*Z* = 1
*M**_r_* = 1198.76	*F*(000) = 610
Triclinic, *P*1	*D*_x_ = 1.722 Mg m^−^^3^*D*_m_ = 1.722 Mg m^−^^3^*D*_m_ measured by not measured
Hall symbol: -P 1	Mo *K*α radiation, λ = 0.71073 Å
*a* = 8.2943 (4) Å	Cell parameters from 3653 reflections
*b* = 11.0232 (5) Å	θ = 4.4–25°
*c* = 13.6139 (7) Å	µ = 0.81 mm^−^^1^
α = 102.690 (4)°	*T* = 292 K
β = 107.282 (4)°	Block, yellow
γ = 90.459 (4)°	0.32 × 0.27 × 0.23 mm
*V* = 1155.9 (1) Å^3^	

### Data collection

**Table:**

Oxford Diffraction Gemini R Ultra CCD diffractometer	4041 independent reflections
Radiation source: fine-focus sealed tube	2933 reflections with *I* > 2σ(*I*)
graphite	*R*_int_ = 0.028
ω scans	θ_max_ = 25.0°, θ_min_ = 4.4°
Absorption correction: multi-scan (*CrysAlis RED*; Oxford Diffraction, 2006)	*h* = −9→9
*T*_min_ = 0.771, *T*_max_ = 0.829	*k* = −13→11
7153 measured reflections	*l* = −14→16

### Refinement

**Table:**

Refinement on *F*^2^	Primary atom site location: structure-invariant direct methods
Least-squares matrix: full	Secondary atom site location: difference Fourier map
*R*[*F*^2^ > 2σ(*F*^2^)] = 0.044	Hydrogen site location: inferred from neighbouring sites
*wR*(*F*^2^) = 0.111	H atoms treated by a mixture of independent and constrained refinement
*S* = 1.00	*w* = 1/[σ^2^(*F*_o_^2^) + (0.0638*P*)^2^] where *P* = (*F*_o_^2^ + 2*F*_c_^2^)/3
4041 reflections	(Δ/σ)_max_ = 0.002
378 parameters	Δρ_max_ = 0.97 e Å^−^^3^
168 restraints	Δρ_min_ = −1.00 e Å^−^^3^

### Fractional atomic coordinates and isotropic or equivalent isotropic displacement parameters (Å^2^)

**Table:**

	*x*	*y*	*z*	*U*_iso_*/*U*_eq_	
C1	−0.0231 (5)	0.3815 (4)	0.6428 (3)	0.0304 (9)	
H1	0.0452	0.3635	0.7049	0.037*	
C2	−0.1233 (6)	0.4828 (4)	0.6499 (3)	0.0346 (10)	
H2	−0.1214	0.5307	0.7157	0.041*	
C3	−0.2237 (5)	0.5105 (4)	0.5595 (3)	0.0336 (9)	
H3	−0.2911	0.5774	0.5631	0.040*	
C4	−0.2245 (5)	0.4375 (4)	0.4614 (3)	0.0286 (8)	
C5	−0.3250 (5)	0.4543 (4)	0.3592 (3)	0.0299 (8)	
C6	−0.3182 (5)	0.3757 (4)	0.2679 (3)	0.0311 (9)	
C7	−0.2138 (5)	0.2745 (4)	0.2631 (3)	0.0277 (8)	
C8	−0.1991 (5)	0.1923 (4)	0.1726 (3)	0.0344 (9)	
H8	−0.2600	0.2020	0.1058	0.041*	
C9	−0.0946 (6)	0.0974 (4)	0.1829 (3)	0.0369 (10)	
H9	−0.0838	0.0422	0.1233	0.044*	
C10	−0.0049 (5)	0.0841 (4)	0.2834 (3)	0.0325 (9)	
H10	0.0650	0.0189	0.2895	0.039*	
C11	−0.1165 (5)	0.2559 (4)	0.3627 (3)	0.0247 (8)	
C12	−0.1215 (5)	0.3373 (3)	0.4607 (3)	0.0249 (8)	
C13	−0.4998 (5)	0.5157 (4)	0.2329 (3)	0.0322 (9)	
C14	−0.6334 (5)	0.5801 (4)	0.1703 (3)	0.0356 (9)	
C15	−0.6963 (6)	0.6831 (4)	0.2208 (4)	0.0438 (11)	
H15	−0.6518	0.7137	0.2935	0.053*	
C16	−0.8264 (7)	0.7403 (5)	0.1618 (4)	0.0546 (13)	
H16	−0.8696	0.8091	0.1957	0.066*	
C17	−0.8916 (7)	0.6973 (5)	0.0553 (4)	0.0606 (14)	
H17	−0.9787	0.7365	0.0167	0.073*	
C18	−0.8286 (7)	0.5960 (6)	0.0052 (4)	0.0619 (15)	
H18	−0.8723	0.5671	−0.0677	0.074*	
C19	−0.7008 (7)	0.5364 (5)	0.0621 (4)	0.0511 (13)	
H19	−0.6599	0.4668	0.0276	0.061*	
C20	0.3815 (5)	−0.0284 (4)	0.6442 (3)	0.0269 (8)	
C21	0.5087 (5)	−0.0973 (4)	0.6896 (3)	0.0328 (9)	
H21	0.5419	−0.1637	0.6466	0.039*	
C22	0.5884 (5)	−0.0697 (4)	0.7980 (3)	0.0368 (9)	
C23	0.5475 (5)	0.0299 (4)	0.8637 (3)	0.0360 (9)	
H23	0.6050	0.0499	0.9358	0.043*	
C24	0.4202 (5)	0.0997 (4)	0.8213 (3)	0.0323 (9)	
C25	0.3257 (5)	0.0739 (4)	0.7106 (3)	0.0265 (8)	
C26	0.2996 (5)	−0.0712 (4)	0.5268 (3)	0.0271 (8)	
N1	−0.0218 (4)	0.3109 (3)	0.5517 (2)	0.0254 (7)	
N2	−0.0149 (4)	0.1606 (3)	0.3706 (2)	0.0250 (6)	
N3	−0.4378 (4)	0.5429 (3)	0.3369 (3)	0.0324 (8)	
N4	−0.4312 (4)	0.4167 (3)	0.1875 (3)	0.0321 (8)	
H4	−0.4538	0.3850	0.1209	0.039*	
N5	0.7161 (5)	−0.1487 (4)	0.8424 (3)	0.0484 (9)	
N6	0.3854 (5)	0.2056 (4)	0.8942 (3)	0.0446 (9)	
O1	0.1485 (3)	−0.0397 (2)	0.4865 (2)	0.0305 (5)	
O2	0.3743 (4)	−0.1379 (3)	0.4735 (2)	0.0459 (8)	
O3	0.2036 (4)	0.1369 (3)	0.6762 (2)	0.0339 (5)	
O4	0.7338 (5)	−0.2469 (4)	0.7867 (3)	0.0650 (9)	
O5	0.8033 (5)	−0.1117 (4)	0.9352 (3)	0.0691 (10)	
O6	0.4661 (6)	0.2254 (4)	0.9869 (3)	0.0813 (11)	
O7	0.2851 (6)	0.2779 (4)	0.8644 (3)	0.0857 (10)	
OW1	0.3241 (4)	0.2215 (3)	0.5222 (3)	0.0345 (5)	
Co	0.09090 (7)	0.14151 (5)	0.52610 (4)	0.0264 (2)	
H1WA	0.362 (7)	0.285 (6)	0.566 (5)	0.064 (18)*	
H1WB	0.407 (7)	0.184 (5)	0.523 (4)	0.058 (16)*	

### Atomic displacement parameters (Å^2^)

**Table:**

	*U*^11^	*U*^22^	*U*^33^	*U*^12^	*U*^13^	*U*^23^
C1	0.034 (2)	0.031 (2)	0.0251 (15)	0.0072 (17)	0.0071 (17)	0.0072 (14)
C2	0.041 (3)	0.031 (2)	0.0312 (16)	0.0091 (18)	0.0129 (18)	0.0042 (17)
C3	0.032 (2)	0.031 (2)	0.0381 (15)	0.0114 (19)	0.0118 (18)	0.0079 (15)
C4	0.027 (2)	0.0249 (19)	0.0326 (12)	0.0062 (16)	0.0055 (16)	0.0089 (14)
C5	0.026 (2)	0.027 (2)	0.0351 (13)	0.0054 (16)	0.0042 (16)	0.0115 (14)
C6	0.027 (2)	0.035 (2)	0.0308 (14)	0.0071 (17)	0.0025 (16)	0.0133 (14)
C7	0.024 (2)	0.0286 (19)	0.0279 (13)	0.0026 (15)	0.0021 (15)	0.0094 (14)
C8	0.036 (3)	0.040 (2)	0.0254 (16)	0.0039 (18)	0.0045 (18)	0.0088 (15)
C9	0.040 (3)	0.041 (2)	0.0267 (14)	0.0078 (19)	0.0101 (18)	0.0022 (17)
C10	0.029 (2)	0.037 (2)	0.0295 (13)	0.0080 (19)	0.0082 (17)	0.0038 (15)
C11	0.020 (2)	0.0269 (19)	0.0264 (12)	0.0036 (15)	0.0042 (15)	0.0091 (13)
C12	0.021 (2)	0.0230 (18)	0.0279 (12)	0.0032 (15)	0.0026 (15)	0.0069 (13)
C13	0.031 (2)	0.030 (2)	0.0349 (14)	0.0058 (16)	0.0057 (16)	0.0121 (16)
C14	0.030 (2)	0.036 (2)	0.0398 (16)	0.0072 (17)	0.0048 (16)	0.0158 (16)
C15	0.044 (3)	0.036 (2)	0.049 (2)	0.011 (2)	0.008 (2)	0.0137 (18)
C16	0.048 (3)	0.047 (3)	0.072 (2)	0.021 (2)	0.014 (2)	0.025 (2)
C17	0.046 (3)	0.070 (3)	0.069 (2)	0.023 (3)	0.005 (2)	0.041 (2)
C18	0.058 (4)	0.074 (4)	0.045 (2)	0.015 (3)	−0.005 (2)	0.024 (2)
C19	0.050 (3)	0.059 (3)	0.0401 (16)	0.018 (2)	0.005 (2)	0.0139 (19)
C20	0.019 (2)	0.029 (2)	0.0318 (13)	0.0034 (15)	0.0040 (13)	0.0095 (14)
C21	0.025 (2)	0.035 (2)	0.0384 (15)	0.0094 (17)	0.0063 (16)	0.0134 (16)
C22	0.024 (2)	0.047 (2)	0.0400 (16)	0.0079 (18)	0.0040 (16)	0.0203 (16)
C23	0.027 (2)	0.051 (2)	0.0278 (19)	0.0026 (18)	−0.0005 (17)	0.0160 (16)
C24	0.025 (2)	0.042 (2)	0.0263 (13)	0.0014 (17)	0.0010 (14)	0.0089 (14)
C25	0.022 (2)	0.031 (2)	0.0252 (13)	0.0047 (15)	0.0029 (14)	0.0104 (13)
C26	0.0219 (17)	0.0223 (19)	0.0328 (14)	0.0051 (15)	0.0040 (12)	0.0035 (14)
N1	0.0257 (17)	0.0237 (14)	0.0258 (11)	0.0052 (13)	0.0052 (13)	0.0070 (11)
N2	0.0223 (16)	0.0260 (15)	0.0258 (10)	0.0044 (12)	0.0052 (12)	0.0071 (11)
N3	0.032 (2)	0.0288 (17)	0.0350 (13)	0.0090 (15)	0.0051 (15)	0.0109 (13)
N4	0.033 (2)	0.0336 (18)	0.0272 (14)	0.0092 (15)	0.0014 (14)	0.0112 (13)
N5	0.034 (2)	0.062 (2)	0.0523 (18)	0.0166 (19)	0.0058 (15)	0.0300 (15)
N6	0.041 (2)	0.057 (2)	0.0267 (13)	0.0099 (17)	0.0015 (15)	0.0037 (14)
O1	0.0280 (10)	0.0323 (9)	0.0278 (9)	0.0142 (9)	0.0037 (8)	0.0059 (8)
O2	0.0315 (15)	0.0534 (16)	0.0404 (14)	0.0173 (13)	0.0048 (12)	−0.0062 (12)
O3	0.0321 (11)	0.0361 (11)	0.0274 (8)	0.0156 (9)	0.0014 (8)	0.0050 (8)
O4	0.055 (2)	0.0667 (19)	0.0686 (19)	0.0307 (17)	0.0039 (16)	0.0270 (15)
O5	0.059 (2)	0.084 (2)	0.0569 (17)	0.0309 (18)	−0.0046 (14)	0.0289 (16)
O6	0.092 (2)	0.092 (2)	0.0308 (12)	0.0423 (19)	−0.0108 (15)	−0.0076 (15)
O7	0.0906 (19)	0.0901 (18)	0.0420 (14)	0.0503 (15)	−0.0127 (14)	−0.0113 (14)
OW1	0.0268 (11)	0.0354 (13)	0.0367 (13)	0.0091 (10)	0.0058 (10)	0.0039 (11)
Co	0.0251 (3)	0.0267 (3)	0.0236 (3)	0.0104 (2)	0.0015 (2)	0.0059 (2)

### Geometric parameters (Å, °)

**Table:**

C1—N1	1.314 (5)	C17—C18	1.369 (8)
C1—C2	1.401 (5)	C17—H17	0.9300
C1—H1	0.9300	C18—C19	1.379 (6)
C2—C3	1.365 (6)	C18—H18	0.9300
C2—H2	0.9300	C19—H19	0.9300
C3—C4	1.398 (6)	C20—C21	1.379 (5)
C3—H3	0.9300	C20—C25	1.453 (5)
C4—C12	1.402 (5)	C20—C26	1.503 (6)
C4—C5	1.446 (5)	C21—C22	1.389 (6)
C5—C6	1.366 (6)	C21—H21	0.9300
C5—N3	1.377 (5)	C22—C23	1.368 (6)
C6—N4	1.376 (5)	C22—N5	1.454 (5)
C6—C7	1.420 (5)	C23—C24	1.373 (5)
C7—C8	1.398 (6)	C23—H23	0.9300
C7—C11	1.416 (5)	C24—C25	1.440 (5)
C8—C9	1.368 (6)	C24—N6	1.448 (5)
C8—H8	0.9300	C25—O3	1.265 (4)
C9—C10	1.389 (6)	C26—O2	1.220 (5)
C9—H9	0.9300	C26—O1	1.294 (4)
C10—N2	1.322 (5)	N4—H4	0.8600
C10—H10	0.9300	N5—O4	1.215 (5)
C11—N2	1.356 (5)	N5—O5	1.229 (5)
C11—C12	1.447 (5)	N6—O7	1.202 (5)
C12—N1	1.363 (5)	N6—O6	1.209 (5)
C13—N3	1.319 (5)	OW1—H1WA	0.81 (6)
C13—N4	1.349 (5)	OW1—H1WB	0.80 (5)
C13—C14	1.475 (5)	Co—N1	2.102 (3)
C14—C19	1.383 (6)	Co—N2	2.095 (3)
C14—C15	1.383 (6)	Co—O1	2.050 (2)
C15—C16	1.388 (6)	Co—O1^i^	2.216 (3)
C15—H15	0.9300	Co—O3	1.991 (3)
C16—C17	1.359 (8)	Co—OW1	2.139 (3)
C16—H16	0.9300		
			
N1—C1—C2	122.5 (4)	C21—C20—C26	116.4 (3)
N1—C1—H1	118.8	C25—C20—C26	123.5 (3)
C2—C1—H1	118.8	C20—C21—C22	121.5 (4)
C3—C2—C1	119.4 (4)	C20—C21—H21	119.3
C3—C2—H2	120.3	C22—C21—H21	119.3
C1—C2—H2	120.3	C23—C22—C21	121.1 (4)
C2—C3—C4	119.4 (4)	C23—C22—N5	119.6 (4)
C2—C3—H3	120.3	C21—C22—N5	119.3 (4)
C4—C3—H3	120.3	C22—C23—C24	118.9 (4)
C3—C4—C12	117.8 (3)	C22—C23—H23	120.6
C3—C4—C5	125.9 (3)	C24—C23—H23	120.6
C12—C4—C5	116.3 (3)	C23—C24—C25	123.5 (4)
C6—C5—N3	110.5 (3)	C23—C24—N6	116.4 (4)
C6—C5—C4	121.0 (3)	C25—C24—N6	120.0 (3)
N3—C5—C4	128.5 (4)	O3—C25—C24	121.1 (3)
C5—C6—N4	105.3 (3)	O3—C25—C20	124.0 (3)
C5—C6—C7	124.8 (3)	C24—C25—C20	114.9 (3)
N4—C6—C7	129.9 (4)	O2—C26—O1	122.3 (4)
C8—C7—C11	117.7 (3)	O2—C26—C20	119.4 (3)
C8—C7—C6	127.5 (4)	O1—C26—C20	118.2 (3)
C11—C7—C6	114.8 (3)	C1—N1—C12	118.8 (3)
C9—C8—C7	119.6 (4)	C1—N1—Co	127.4 (2)
C9—C8—H8	120.2	C12—N1—Co	113.2 (2)
C7—C8—H8	120.2	C10—N2—C11	119.2 (3)
C8—C9—C10	119.4 (4)	C10—N2—Co	127.0 (2)
C8—C9—H9	120.3	C11—N2—Co	113.5 (2)
C10—C9—H9	120.3	C13—N3—C5	104.8 (3)
N2—C10—C9	122.7 (4)	C13—N4—C6	107.2 (3)
N2—C10—H10	118.7	C13—N4—H4	126.4
C9—C10—H10	118.7	C6—N4—H4	126.4
N2—C11—C7	121.5 (3)	O4—N5—O5	123.0 (4)
N2—C11—C12	116.9 (3)	O4—N5—C22	119.2 (4)
C7—C11—C12	121.7 (3)	O5—N5—C22	117.9 (4)
N1—C12—C4	122.0 (3)	O7—N6—O6	119.1 (4)
N1—C12—C11	116.5 (3)	O7—N6—C24	121.8 (4)
C4—C12—C11	121.5 (3)	O6—N6—C24	118.9 (3)
N3—C13—N4	112.1 (3)	C26—O1—Co	120.4 (3)
N3—C13—C14	125.6 (4)	C26—O1—Co^i^	125.8 (2)
N4—C13—C14	122.2 (4)	Co—O1—Co^i^	100.87 (10)
C19—C14—C15	119.4 (4)	C25—O3—Co	127.0 (2)
C19—C14—C13	120.8 (4)	Co—OW1—H1WA	116 (4)
C15—C14—C13	119.8 (4)	Co—OW1—H1WB	124 (4)
C14—C15—C16	119.4 (5)	H1WA—OW1—H1WB	102 (5)
C14—C15—H15	120.3	O3—Co—O1	87.05 (10)
C16—C15—H15	120.3	O3—Co—N2	175.08 (14)
C17—C16—C15	120.9 (5)	O1—Co—N2	96.09 (11)
C17—C16—H16	119.5	O3—Co—N1	98.70 (11)
C15—C16—H16	119.5	O1—Co—N1	167.77 (12)
C16—C17—C18	119.7 (4)	N2—Co—N1	78.95 (12)
C16—C17—H17	120.1	O3—Co—OW1	88.76 (12)
C18—C17—H17	120.1	O1—Co—OW1	95.00 (13)
C17—C18—C19	120.5 (5)	N2—Co—OW1	87.20 (12)
C17—C18—H18	119.7	N1—Co—OW1	95.91 (13)
C19—C18—H18	119.7	O3—Co—O1^i^	95.02 (11)
C18—C19—C14	120.0 (5)	O1—Co—O1^i^	79.13 (10)
C18—C19—H19	120.0	N2—Co—O1^i^	89.30 (11)
C14—C19—H19	120.0	N1—Co—O1^i^	89.56 (11)
C21—C20—C25	119.9 (4)	OW1—Co—O1^i^	172.82 (12)

Symmetry codes: (i) −*x*, −*y*, −*z*+1.

### Hydrogen-bond geometry (Å, °)

**Table:**

*D*—H···*A*	*D*—H	H···*A*	*D*···*A*	*D*—H···*A*
N4—H4···O6^ii^	0.86	2.17	2.948 (5)	150
OW1—H1WA···N3^iii^	0.81 (6)	2.03 (6)	2.831 (5)	172 (5)
OW1—H1WB···O2^iv^	0.80 (5)	1.88 (5)	2.662 (4)	165 (5)

Symmetry codes: (ii) *x*−1, *y*, *z*−1; (iii) −*x*, −*y*+1, −*z*+1; (iv) −*x*+1, −*y*, −*z*+1.

## References

[bb1] Brandenburg, K. (1999). *DIAMOND* Crystal Impact GbR, Bonn, Germany.

[bb2] Che, G.-B., Liu, C.-B., Liu, B., Wang, Q.-W. & Xu, Z.-L. (2008). *CrystEngComm*, **10**, 184–191.

[bb3] Liu, D.-M., Li, X.-Y., Wang, X.-C., Li, C.-X. & Liu, C.-B. (2009). *Acta Cryst.* E**65**, o1308. 10.1107/S1600536809017498 PMC2969696 21583165

[bb4] Oxford Diffraction (2006). *CrysAlis CCD* and *CrysAlis RED* Oxford Diffraction Ltd, Abingdon, Oxfordshire, England.

[bb5] Sheldrick, G. M. (2008). *Acta Cryst.* A**64**, 112–122. 10.1107/S0108767307043930 18156677

[bb6] Steck, E. A. & Day, A. R. (1943). *J. Am. Chem. Soc.* **65**, 452–456.

